# A Hybrid Particle Swarm Optimization Algorithm with Dynamic Adjustment of Inertia Weight Based on a New Feature Selection Method to Optimize SVM Parameters

**DOI:** 10.3390/e25030531

**Published:** 2023-03-19

**Authors:** Jing Wang, Xingyi Wang, Xiongfei Li, Jiacong Yi

**Affiliations:** 1 School of Media Science & School of Journalism, Northeast Normal University, Jingyue, Changchun 130117, China; 2 Key Laboratory of Symbolic Computation and Knowledge Engineering of Ministry of Education, Jilin University, Qianjin, Changchun 130012, China; 3 College of Computer Science and Technology, Jilin University, Qianjin, Changchun 130012, China

**Keywords:** optimization, SVM, FS-score, particle swarm optimization, feature selection

## Abstract

Support vector machine (SVM) is a widely used and effective classifier. Its efficiency and accuracy mainly depend on the exceptional feature subset and optimal parameters. In this paper, a new feature selection method and an improved particle swarm optimization algorithm are proposed to improve the efficiency and the classification accuracy of the SVM. The new feature selection method, named Feature Selection-score (FS-score), performs well on data sets. If a feature makes the class external sparse and the class internal compact, its FS-score value will be larger and the probability of being selected will be greater. An improved particle swarm optimization model with dynamic adjustment of inertia weight (DWPSO-SVM) is also proposed to optimize the parameters of the SVM. By improving the calculation method of the inertia weight of the particle swarm optimization (PSO), inertia weight can decrease nonlinearly with the number of iterations increasing. In particular, the introduction of random function brings the inertia weight diversity in the later stage of the algorithm and the global searching ability of the algorithm to avoid falling into local extremum. The experiment is performed on the standard UCI data sets whose features are selected by the FS-score method. Experiments demonstrate that our algorithm achieves better classification performance compared with other state-of-the-art algorithms.

## 1. Introduction

Support vector machine (SVM) is one of the most popular machine learning methods, which is based on the statistical learning theory and the structural risk minimization principle [1]. Moreover, it has unique advantages in solving practical problems, such as small sample data sets as well as nonlinear and high-dimensional pattern recognition [2,3,4]. In SVM, the kernel function selection is a key part for nonlinear problems. The Radial Basis Function (RBF), as the most common kernel function, has the characteristics of fewer optimized parameters and better classification performance in terms of accuracy and stability, especially for high-dimensional data. However, the classification effect of the SVM is related to two factors. The first one is the quality of the input data set. The greater the degree of data set differentiation, the better the algorithm effects. The second one is the value of the error penalty parameters and the kernel parameters. In order to achieve the best classification effect and the maximum generalization of the SVM, many researchers have proposed different solutions to solve the two issues above.

One of the most widespread methods for selecting these two key parameters is using the grid algorithm to traverse and compare iteratively. This method can achieve relatively good results, but it is time-consuming [5]. Therefore, some researchers proposed using the meta-heuristic algorithm to find the best parameter value within the specified range [6,7,8,9]. The most commonly used meta-heuristic algorithms are the genetic algorithms. They perform well in solving large-scale nonlinear problems [10,11]. However, they are easy to fall into the local optimal value and fail to find the global optimal solution [12,13,14], since the algorithm follows the natural law of information exchange and evolution among populations. Another meta-heuristic algorithm used to find the optimal parameters is particle swarm optimization (PSO), which mimics the information exchange between birds in searching for food to find the optimal result in the population. Each iteration of the algorithm will be adjusted according to the results of the previous iteration, which greatly improves the efficiency. Compared with the cross-mutation of the genetic algorithm, it is more targeted, which weakens its randomness. However, the PSO algorithm is as easy as the genetic algorithm to fall into local optimum, resulting in large errors in the results [15,16]. Mafarja et al. proposed the Binary Dragonfly Algorithm for minimizing all features using the wrapper feature selection algorithms to enhance the classification accuracy [17]. By taking a mutational approach and scanning the area surrounding the explorers, Binary Al-Biruni Earth Radius Optimization is able to deliver cutting-edge exploration capabilities. It ensures diversity and thorough exploration by shuffling the order of responses between repetitions [18]. Khafaga et al. proposed the Binary Dipper Throated Optimization (bDTO) method to optimize the weighted ensemble model by the DTO algorithm. Once the significant features are selected, the optimized weighted ensemble model is employed to predict the gain values of metamaterial antenna [19]. The Binary Cat Swarm Optimization (BCSO) algorithm includes two major modes: tracing and seeking modes. However, the traditional BCSO has the chance of becoming trapped in the local optima [20].

Many scholars have improved the PSO algorithm in recent years. The most common method is the change of inertia weight, which can improve the global search ability of the algorithm [21,22,23]. Inertial weight *w* is a very important parameter in the PSO algorithm, which plays an important role in the global searching ability of the algorithm. It increases the diversity of the population and speeds up the convergence speed. Clerc proposed that the value of *w* was 0.729 [24,25]. Trelea suggested that *w* should be 0.6 [14,26]. The smaller *w* is, the stronger the local search ability of the algorithm is and, consequently, the faster the algorithm converges. However, it is easier to fall into local convergence, and its search ability in new regions is relatively deficient. The larger *w* is, the stronger the global search ability of the algorithm is, and the more diversity the population reflects. However, it is easy to miss the optimal point when *w* is large, thus leading to non-convergence. Therefore, it is of importance to adjust the value of *w* dynamically to obtain the right value at different times. At the earliest, Shi and Eberhart proposed adjusting the inertia weight *w* by the way of linear decline [27]. In their view, the minimum value of *w* is taken as the bottom line, and the value of *w* decreases linearly from the maximum to the minimum with the number of iterations. As the actual optimization process is nonlinear and complex, the linear decline way is not in line with the actual rules of optimization. It did not consider the situation of all the particles in the population and had no strong adjustment, so it is easy to fall into local optimum and cannot obtain better results.

Later, Shi and Eberhart introduced the random inertia weighting strategy, which changed the value of *w* by introducing a random function [27]. Some scholars put forward the nonlinear decreasing method to adjust the inertia weight, such as the decreasing inertia weight adjustment strategy in the form of a parabola by Chatterjee and Siarry [28]. Jiang et al. used the change of cosine function to realize the nonlinear decreasing strategy [24]. This nonlinear decreasing method improves the searching efficiency of the algorithm to a certain extent. It can overcome the premature convergence of the algorithm and reduce the probability of falling into local extreme value to a certain extent [29,30,31]. Lu et al. introduced the concept of personalized inertia weight. This method judges the current position of each particle by comparing its fitness with the average fitness of the population. Based on the state, the inertia weight of a particle with a better position is reduced, and the particle is focused on the development of the current region. For the particles in poor positions, the inertia weight of the particle is increased, causing the particle to focus on potential area exploration [32].

In the above algorithms, the values of *w* in the early stage are large, so they have a strong global search ability. However, with the decrease of *w* in the later stage, the algorithm easily falls into the local optimum, resulting in the loss of population diversity, and the convergence speed of the algorithm will also slow down. Therefore, in the later part of the algorithm, the *w* value will still become very small. In this way, the group does not have diversity, it is easy to fall into a local extreme value, and it does not have a strong global search ability. At the same time, the cosine function cannot be adjusted in time for specific situations. The flexibility of the equation is poor [33,34,35].

The application of SVM relies on training in corresponding data sets to build corresponding models. The rapid development of modern society leads to the explosive growth of all kinds of information and data [36]. The growth is not only in quantity but also in more and more diversified forms of data, such as text documents, pictures and gene sequences [37]. Faced with such massive data and complex forms of expression, it is impossible to carry out the practical application without filter and process. At the same time, the relationship and characteristics of massive data in modern society also have wider application space [38,39]. Therefore, data screening and dimension reduction of complex data are becoming more and more important, while the feature selection method is the key to achieving the above applications. Feature selection is a widely used data processing method, which selects the conforming features and excludes the non-conforming features according to the set evaluation criteria [40]. New data sets are obtained by the feature selection of the original data sets, which can improve the accuracy and efficiency of the algorithm in classification, regression, prediction and other tasks. It makes the models we generate more accurate and easier to understand.

The main methods of feature selection include wrapping, filtering and embedded methods. Most feature selection methods are derived and improved from these three methods. Some scholars use SVM classifier for feature selection. When redundant features or interference features exist, the performance of the classifier will be significantly reduced. In addition, forward feature selection and backward feature selection are commonly used in feature selection by classifier. Different feature selection methods should be applied to data sets with different characteristics.

In order to further improve the accuracy of SVM classification, we intend to solve it from two different perspectives. First, an efficient new multi-class feature selection method is proposed, which is simple and intuitive. When using the hybrid model of particle swarm optimization support vector machine (PSO-VM) to evaluate the proposed feature selection method, not only the accuracy is greatly improved but also the training time is shortened, and a good effect is achieved. Then, to address the drawback of the elementary PSO algorithm where it is easy to fall into local optimum, a method based on the dynamic change of the inertia weight is proposed to optimize the PSO, which can enhance the global searching ability of PSO and expand the diversity of the population. The improved PSO algorithm is used to optimize the parameters of SVM, which improves the performance of the model and enhances the generalization ability of the model. In summary, the main contributions of this paper are presented as follows:

1. A new feature selection method is proposed. In the formula, the numerator is the sum of the mean values of the variances between classes, and the denominator is the dispersion coefficient to measure the dispersion degree of each eigenvalue within the class. When the numerator is larger and the denominator is smaller, the gap between classes will be larger and the gap within each class will be smaller. The discretization coefficient can describe the differences between classes more accurately and have a better performance than other ways. The proposed feature selection method can improve the classification accuracy and shorten the training time of the classifier.
2. An improved method for the inertia factor in PSO is proposed, which dynamically changes the inertia factor. We use the logarithmic function and random number to improve the changing process. It not only ensures that the inertia weight can decrease nonlinearly but also meets the necessary conditions for its convergence. In addition, a random function is introduced to ensure that the algorithm has a strong search ability in the early stage, and it will not be premature in the later stage. This algorithm can improve the accuracy of the search.
3. The proposed feature selection method is combined with the proposed Dynamic Weighted Particle Swarm Optimization (DWPSO) algorithm to improve the classification accuracy of the SVM and shorten the whole experiment time.

This paper is organized as follows. Section 2 introduces the method of the feature selection. Section 3 makes a brief introduction to SVM, PSO and introduces the DWPSO-SVM model. Section 4 describes the experiments performed and the obtained results. Section 5 discusses the main conclusions and future work.

## 2. Feature Selection

In this section, the new feature selection method called FS-score is detailed. By using this method, the key features can be selected, and the redundant or irrelevant features can be eliminated so as to build a better representation of data and improve both the accuracy and computation efficiency of classification algorithms.

The feature selection method selects the most effective feature for classification. Multi-classification feature selection refers to the feature selection on the data sets with multiple classes. To solve the problem of low classification accuracy of multi-classes and multi-feature data sets, the method to improve the efficiency of the algorithm and shorten the running time is proposed. The feature selection method is described as follows:

Given data set $Q\in R^{m},\;x_{k}$ is the *k*-th sample in the data set, where k=1,2,3…n(n⩾2), and $n_{j}$ is the number of samples of class *j*. Then, the FS-score value of the *i*-th feature in the data set is calculated below.

(1) $$F_{i}=\frac{\sum_{j=1}^{p}{\left({\bar{x}}_{i}^{j}-\bar{x_{i}}\right)}^{2}}{q_{i}}$$

(2) $$q_{i}=\sum_{j=1}^{p}\frac{\sqrt{\sum_{k=1}^{n_{j}}({\left(x_{k}\right)}_{i}^{j}-{\bar{x}}_{i}^{j}{)}^{2}}}{\bar{x_{j}}}$$

In Equations (1) and (2), ${\bar{x}}_{i}^{j}$ is the average characteristic value of the *i*-th feature in the *j*-th class. $\bar{x_{i}}$ is the average characteristic value of the *i*-th feature in the whole data set. *p* is the total number of classes. ${\left(x_{k}\right)}_{i}^{j}$ is the characteristic value, where *j*, *k* and *i* refer to the *j*-th class, the *k*-th sample and the *i*-th feature, respectively.

The proposed feature selection method is based on the value of $F_{i}$. In Equation (1), the numerator is the sum of the mean values of the variances between classes, and the denominator is the dispersion coefficient to measure the dispersion degree of each eigenvalue within the class. The discretization coefficient can be used to describe the differences between classes more accurately. When the numerator is larger and the denominator is smaller, the gap between classes will be bigger and the gap within classes will be smaller. This feature will play an important role in the classification, and the discrimination ability of the feature will be stronger. Therefore, when the $F_{i}$ value is larger, the probability of this feature being selected is higher.

The principle upheld by the feature selection method is that the larger the gap between categories and the smaller the gap within categories, the better the classification effect will be. In many other methods, numerators represent the approximate sum of distances between classes. If there is an extreme value, it is very easy to cause a too large or too small result, which will cause interference in the process of feature selection. In the FS-score method, the numerator is the sum of the mean values of various inter-class variances; it can avoid the interference caused by extreme values on feature selection. The denominator is the discrete coefficient to measure the dispersion degree of each feature value within the class. Using the discrete coefficient will make the description of the difference between classes more accurate and more intuitive.

The algorithm process will be elaborated in order, and the specific process is described in detail in Algorithm 1.
**Algorithm 1** An effective feature extraction method**Input:** The data set $Q\in R^{m}$, the penalty parameters *C*, the kernel parameter γ, the number of iterations *m*.**Procedure:**  1:Value preprocessing: using $g=\frac{g^{\prime }-\min_{a}}{\max_{a}-\min_{a}}$ to scale the eigenvalue. *g* is the scaled value, $g^{\prime }$ is the original value of the feature, $max_{a}$ and $min_{a}$ are the upper and lower bounds of the original eigenvalue, respectively.  2:For the $i^{th}$ sample, i=1,2…,L. Calculating $F_{i}$ using Equations (1) and (2). According to the $F_{i}$ value of features, the features are divided into *u* groups in descending order (u=5∼10).  3:Calculating the accuracy for each group.**Output:** $accuracy\in R^{m}$

## 3. DWPSO-SVM Model

### 3.1. Support Vector Machines

The initial theory of SVM is based on the principle of maximizing the interval and minimizing the structural risk. For practical problems, there are a variety of SVM-derived algorithms, such as the linear SVM, the nonlinear SVM and the least squares SVM. For the traditional dichotomous linear SVM, the hyperplane is used to classify the linearly separable dichotomous data sets [12,41]. Given a dichotomous data set $T\left\{x_{i},y_{i}\right\}\left(i=1,2\ldots n\right)$, where $x_{i}\in R^{n},y_{i}\in \left\{-1,1\right\}$, for the dichotomous linearly separable data set in the $R^{n}$ space, there will be multiple linear classification hyperplanes with the expression $\omega^{T}x+b=0$. The convex quadratic programming model of the linear separable SVM is described as follows:

(3) $$\begin{array}{cc} \min_{\omega ,b}∥\omega ∥*\frac{1}{2} & s.t.\;y_{i}\left(\omega^{T}x_{i}+b\right)\geq 1,i=1,2\ldots n \end{array}$$

According to this constraint condition, the training sample points of the two classes are divided into both sides of the two hyperplanes parallel to the classification hyperplane [11,42]. Sometimes, some sample points of the two classes are too close. In this case, the hyperplane obtained according to Equation (3) cannot properly separate these sample points. To enhance the generalization ability and robustness of the model, the convex quadratic programming problem of the linear SVM is obtained by adding the relaxation variable $\varepsilon_{i}$ and the penalty parameter *C*.

(4) $$\begin{array}{c} \begin{array}{cc} \; & \min_{\omega ,b,\varepsilon }\;\frac{1}{2}{∥\omega ∥}^{2}+C\sum_{i=1}^{n}\varepsilon_{i}\\ \; & s.t.\;y_{i}\left(\omega^{T}x_{i}+b\right)+\varepsilon_{i}\geq 1,i=1,2\ldots n, \end{array} \end{array}$$

where $\varepsilon_{i}\geq 0,\;i=1,2\ldots n$. Equation (4) is a minimization problem that is difficult to solve. It is difficult to find a hyperplane with a large margin while controlling the total amount of relaxation variables [43,44,45]. The penalty parameter *C* is used to balance the two terms. This model can be solved by maximizing the dual Lagrangian formula $L_{D}\left(\alpha \right)$ as follows.

(5) $$\max L_{D}\left(\alpha \right)=\sum_{i}^{n}\alpha_{i}-\sum_{i,j=1}^{m}\alpha_{i}\alpha_{j}y_{i}y_{j}\left(x_{i}*x_{j}\right)*\frac{1}{2},$$

where $0\leq \alpha <C,\;i=1,2\ldots n,\;\sum_{i}^{n}\alpha_{i}y_{i}=0$. ω and *b* can be solved directly. In practical problems, the samples of many data sets cannot be separated linearly. Hence, a nonlinear classifier is needed to separate the data sets through the nonlinear mapping. So, the SVM can solve the problem in a higher-dimensional space. We implement this process by using the following kernel function instead of the dot product.

(6) $$\max L_{D}\left(\alpha \right)=\sum_{i}^{n}\alpha_{i}-\frac{1}{2}\sum_{i,j=1}^{m}\alpha_{i}\alpha_{j}y_{i}y_{j}K\left(x_{i},x_{j}\right),$$

where $0\leq \alpha <C,\;i=1,2\ldots n,\;\sum_{i}^{n}\alpha_{i}y_{i}=0$. α is the Lagrange multiplier. In this paper, the kernel function is chosen to be the radial basis, while the PSO algorithm and the improved PSO algorithm are used to find the best penalty parameter *C* and the kernel parameter γ. The radial basis kernel function is described as follows.

(7) $$K\left(x_{i},x_{j}\right)=\exp(-\gamma ||x_{i}-x_{j}{\left|\right|}^{2})$$

Small samples, high latitude, nonlinearity, and local minimum points are all difficulties that the SVM model excels at handling when using optimization theory. It solves the problems of pattern categorization, dimensionality disaster, and over-learning at the same time, while it has great generalization, global optimal performance and learning ability. It has been extensively utilized and has effectively solved numerous pattern recognition challenges.

Many scholars developed strategies for improving and modifying the SVM model. Osuan et al. devised and implemented a decomposition technique for face detection in 1997. A sequential optimization approach was suggested by Platt in 1998. The purpose of the technique is to supplement the original method with some variables or functions [46]. Suykens et al., for example, presented the LS-SVM approach, which increased support vector sparsity and proposed a sparse approximation strategy for regression issues [47]. In addition, there are more algorithms, such as the dynamic weighted LS-SVM, Huber approximation algorithm and Do Lagrangian multiplier collaborative optimization algorithm. To improve the efficiency of optimization, S S. Keerth proposed the nearest point algorithm to improve the efficiency [48]. Mavroforakis et al. developed an intuitive application for understanding geometric optimization algorithms for the geometric framework of SVM classification issues in terms of geometric methods [49]. In terms of incremental algorithms, Ahmed presented the SVM incremental training method, which selected a small batch of traditional quadratic programming algorithms as increments, allowing the support vector and new samples to be blended for training [50].

Scholars have discovered that the SVM method offers numerous advantages as a result of ongoing in-depth research. It can ensure that the extreme value solution has been found to be the global best solution. This also proves that the SVM method is capable of generalizing unknown samples. Some SVM models and approaches have shown strong promotion ability in numerous application domains and have been widely employed as a result of these advantages. Face detection and recognition, handwritten numbers recognition, text classification, speech recognition, picture recognition, image classification, and the like are some of the most common applications in the field of pattern recognition. Several pattern recognition challenges have been solved satisfactorily.

### 3.2. DWPSO-SVM

The PSO algorithm was put forward by Dr. Eberhart and Dr. Kennedy in 1995. It is a population intelligent algorithm designed by imitating the study on the predation behavior of birds while observing the predation situation [51,52]. In the proposed method, DWPSO is used to find the two best parameters values of the SVM. Scholars have put forward improvement measures to the PSO algorithm from different perspectives; most of them improve the change way of inertia weight, which uses different types of calculation methods to slow down the speed of inertia weight reduction in the later period of the algorithm. However, these methods all have the limitations and shortcomings. In particular, although these algorithms delay the decreasing trend of the inertia factor of the PSO algorithm in the later period, the trend does not change. It is still likely to lead to the local extreme value. The DWPSO-SVM improves the problem that the inertia factor of the PSO algorithm easily falls into local convergence in the later stage of the algorithm, and it achieves a better effect. However, compared with the grid algorithm, the DWPSO-SVM still gives non-optimal parameter values with some probability. However, the efficiency of the time-consuming grid algorithm is poor, and its practical application effect is not good.

Different from the genetic algorithm, the DWPSO-SVM does not need to go through steps such as crossover, mutation and evolution, and it avoids the complex evolutionary operation. It uses mobile search in the whole area, and it can dynamically adjust its search strategy according to the current situation. As all the particles are searching and moving at the same time, it is an efficient parallel search strategy. Moreover, the algorithm is not sensitive to the size of the population, and the number of the population has no obvious effect on the effectiveness of the algorithm [24,27]. Here, we introduce the fundamental PSO algorithm, which has the following formula.

(8) $$\begin{array}{cc} v_{i}= & v_{i}+c_{1}*rand\left(\right)*\left(pbest_{i}-x_{i}\right)\\ \; & +c_{2}*rand\left(\right)*\left(gbest_{i}-x_{i}\right) \end{array}$$

(9) $$x_{i}=x_{i}+v_{i}$$

In Equations (8) and (9), i=1,2…N, *N* denotes the total number of particles in this swarm. $v_{i}$ denotes the velocity of the particle, rand() denotes a random number whose value is between (0, 1), $x_{i}$ denotes the current position of the particle in space, $c_{1}$ and $c_{2}$ are learning factors, and usually, a fixed value will be selected. The maximum value of $v_{i}$ is $V_{max}$. If $v_{i}$ is greater than $V_{max}$ during iteration, then $v_{i}=V_{max}$. $pbest_{i}$ and $gbest_{i}$, respectively, represent the best-known position of the particle itself and the best-known position of particle swarm globally [53,54].

Equations (8) and (9) are called elementary particle swarm equations. The standard particle swarm equation will be introduced below. The specific equation is expressed as follows.

(10) $$\begin{array}{cc} v_{i}= & w*v_{i}+c_{1}*rand\left(\right)*\left(pbest_{i}-x_{i}\right)\\ \; & +c_{2}*rand\left(\right)*\left(gbest_{i}-x_{i}\right) \end{array}$$

Compared with Equation (8), Equation (10) is only modified in the first term. *w* is called the inertial factor, and its value is non-negative. The magnitude of its value will affect the motion of particles in space. When its value is large, the global optimization ability is strong, but the local optimization ability is weak conversely. When its value is small, the global optimization ability is weak, but the local optimization ability is strong. The introduction of *w* has greatly improved the performance of the PSO algorithm, and it can flexibly adjust the global and local search ability according to different actual situations. Equations (9) and (10) refer to the standard PSO algorithm together [55,56].

Next, we present the proposed PSO algorithm based on the improved inertial weight in detail. At the same time, the improved PSO algorithm is used to optimize the parameters of the SVM classifier. This section mainly includes the model elaboration and the algorithm.

A method is proposed about dynamically changing the inertia factor, which can adjust the variation trend of the equation according to the specific situation. The specific method is described as follows:

The natural logarithm is used to control the reduction of inertia weight. rand() is a random generation function, which can increase the diversity of the population in the later stage of the algorithm to avoid premature maturity. The specific formula is presented in detail as follows.

(11) $$\begin{array}{cc} w(t)= & w_{\min}+\left(\frac{w_{\max}-w_{\min}}{2}\right)*\frac{1}{\ln(e+{\left(kz\right)}^{2})}\\ \; & +\frac{\left(w_{\max}-w_{\min}\right)}{2}*rand\left(\right) \end{array}$$

where the value of $w_{\max}$ is 0.9 and $w_{\min}$ is 0.4 [45], z=t/T, *k* is a random number from 0 to 1. In Equation (11), the second part is used to reduce the inertia weight gradually through the logarithmic function $\frac{1}{\ln(e+{\left(kz\right)}^{2})}$. *k* is the regulating factor, which can adjust the decreasing trend of inertia weight. The weight of inertia is large in the early period, and as *z* becomes larger, the first term of the equation becomes smaller. In this case, the third term of the equation adjusts the value of w(t) in time. The value will increase randomly to ensure the diversity of the population and to avoid premature convergence. Therefore, it can be seen from Equation (11) that this algorithm not only ensures that the inertia weight can decrease nonlinearly but also meets the necessary conditions for its convergence. In addition, rand() (random function) is introduced to ensure that the algorithm has a strong search ability in the early stage, and it will not be premature in the later stage, so as to improve the accuracy of the search.

The DWPSO-SVM classifier is utilized to classify the data set after feature selection by using the proposed FS-score method. The kernel selected by the SVM classifier is RBF kernel. The improved particle swarm optimization algorithm is used to find the optimal penalty factor *C* and the parameter γ in the kernel function of SVM. We evaluate the DWPSO-SVM method according to the evaluation indexes. As the data sets are used after feature selection, there is no need to scale the eigenvalues of each sample in the data set. We divide the data sets into three independent parts: training set, monitoring set and test set. The training set is used to train the DWPSO-SVM classifier including the feature selection step. The monitoring set is used to optimize the model such as the number of features and other parameters in the model to avoid overfitting. The test set is used to test the predictive ability of the model independently. The whole algorithm is introduced detailed in Algorithm 2.
**Algorithm 2** The DWPSO algorithm to optimize the parameters of the SVM**Input:** the data set after feature selection $Q\in R^{m}$, the range of the penalty parameter *C*, the range of kernel parameter γ, the number of iterations *m*.**Procedure:**  1:Calculating w(t) using Equation (11) in the PSO algorithm.  2:*Q* is divided into the training set and the testing set, and it is trained on the DWPSO-SVM classifier.  3:For the $i^{th}$ iteration, i=1,2…,m. If the iteration number = *m*, testing begins.  4:Using Equations (12), (13) and (14) to calculate accuracy, TNR and TPR.**Output:** $accuracy\in R^{m}$, TNR, TPR∈[0, 1]

## 4. Experimental Results and Discussions

### 4.1. Experiments Settings

All experiments in this paper are conducted on a personal computer with an Intel 8-generation i5 processor, 2.4 GHz, 8 GB of RAM, and Windows 10. The code runs on Matlab 2016 and is used to experiment with the Libsvm toolkit. All the data sets used in the proposed FS-score method are the standard UCI data sets, while the data sets used in the DWPSO-SVM method are the data sets after being feature selected by the FS-score method on the standard UCI data sets. The values of the two parameters of the RBF kernel function in SVM used in our experiment [57] are *C* in [0, 32,000] and γ in [0, 10]. The characteristics of the data sets, such as the numbers of features and instances, are shown in Table 1. According to the preliminary experimental results, the maximum number of iterations is set to 150, and all experiments converge within 150 generations. The initialization of agents used in the proposed method refers to [58]. Various evaluation criteria are used to evaluate the effectiveness of the proposed approach. The specific evaluation criteria will be described in detail. To verify the effectiveness of our proposed method, the method of averaging ten random trials is used. The original data set is divided into *K* parts. All of the parts except the *K*-th part are trained. The *K*-th part tests the classification effect and returns the value of the classification evaluation index being used. Here, *K* is set to 10.

### 4.2. Evaluation Criteria

In this subsection, the PSO-SVM classifier is used to demonstrate the effectiveness of our proposed feature selection method. The kernel function selected by the classifier is the RBF. The accuracy of classification is used as the criterion to evaluate the effectiveness of the proposed feature method. Its value is calculated according to the confusion matrix. Table 2 indicates the actual category of the samples as well as the predicted category, where 1 denotes positive and −1 denotes negative.

According to the confusion matrix above, the accuracy of the classifier is defined as Equation (12).

(12) $$Accuracy=\frac{TP+TN}{TP+FP+NP+TN}$$

At the same time, the training time of the test samples is considered as a part of the evaluation criteria. When the accuracy difference is within the acceptable range, the fewer features selected, the shorter the training time, and the higher the efficiency of the algorithm.

To evaluate the effectiveness of our proposed DWPSO method, we take advantage of the hybrid matrix mentioned in Table 2 to evaluate our proposed model. Among all the evaluation indexes, accuracy is considered to be the most important metric to evaluate the classification effect. In addition to the accuracy, other corresponding indicators for different types of data sets are also adopted to accurately evaluate the effects. Sensitivity (TPR) and specificity (TNR) are two common evaluation indexes for dichotomous data sets. The specific Equations (13) and (14) are described in detail.

(13) $$TPR=precision=\frac{TP}{TP+FP}$$

(14) $$TNR=\frac{TN}{TN+FP}$$

For binary data sets, the ROC curve and AUC value can also be used to evaluate the classification performance. The ROC curve is the receiver operating characteristic curve. Each point on the curve reflects the sensitivity to the same signal stimulus. The vertical axis of the curve is TPR, and the horizontal axis is FPR. ROC shows the tradeoff between sensitivity and specificity. The closer the curve is to the left and upper boundary of the coordinate axis, the better the classification effect of the classifier will be. The area contained under the ROC curve is the AUC value. Similarly, according to the definition of the ROC curve, the larger the AUC value is, the better the classification effect will be.

### 4.3. Results of Feature Selection with FS-Score

**Figure 1:**
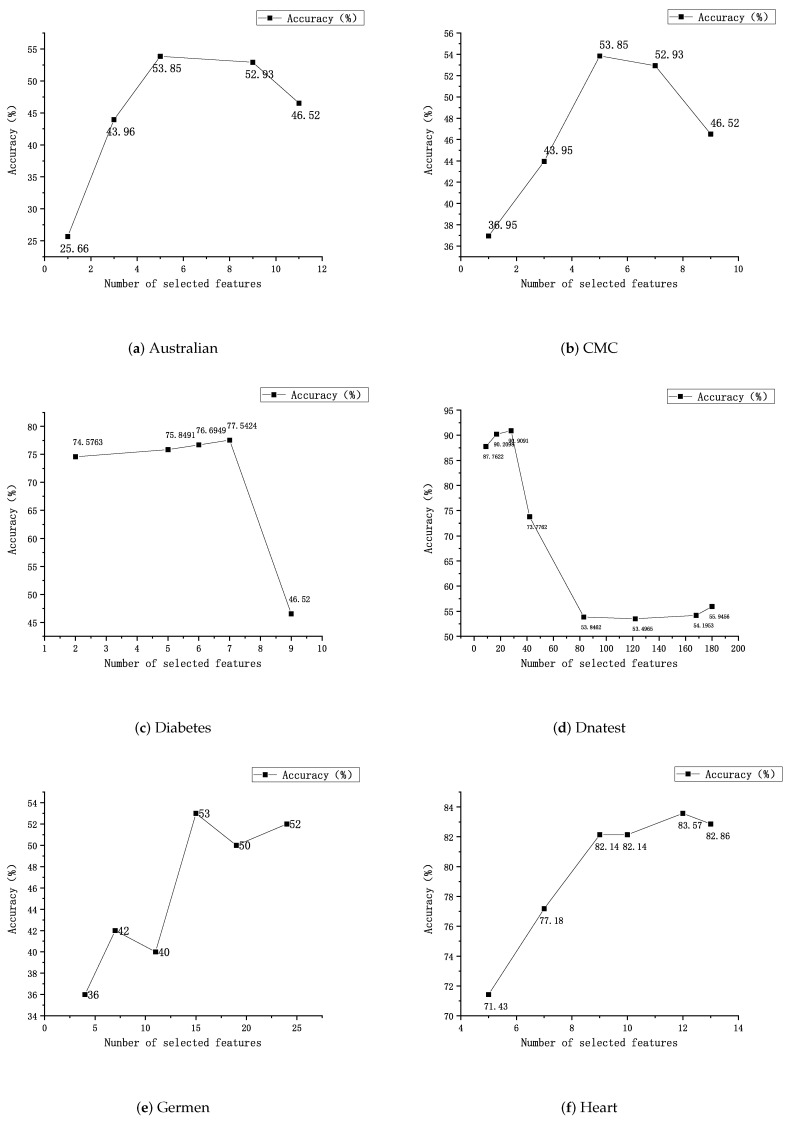

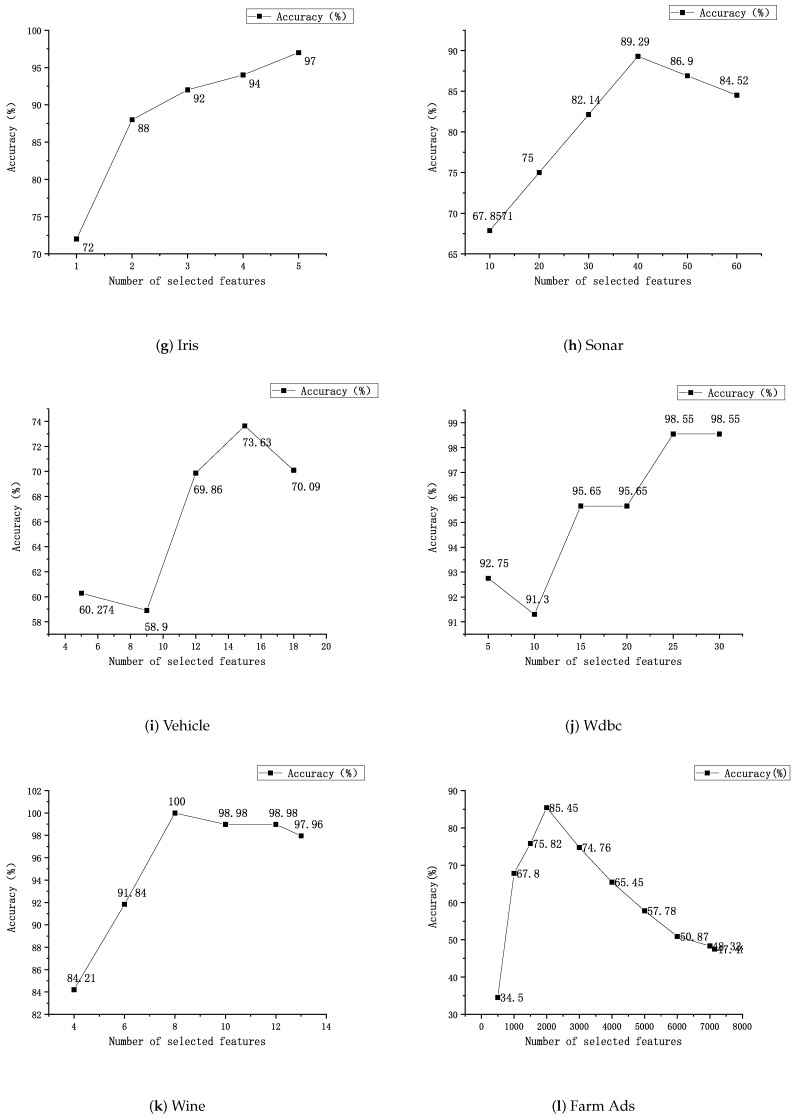

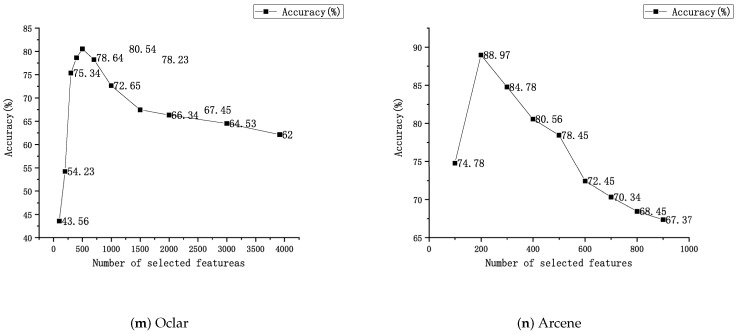
Changes in the accuracy of each data set with the increase in the number of features.

In this part of the experiment, 15 data sets from the standard UCI data set are used (Australia, CMC, Diabetes, Dnatest, Germen, Heart, Iris, Sonar, Vehicle, WDBC, Wine, Air, Farm Ads, Oclar, Arcene). First, FS-scores of the features are calculated, and the features are sorted from the largest to the smallest according to the FS-score value. Then, they are divided into four to ten groups based on the number of features. The first group has at least one feature and at most 15 ranked top by the FS-score. The actual number is determined based on the number of features in the data set. The second group, based on the first group, adds one or more features whose FS-score just stays behind the first group. For example, the data set Iris has a total of four features, and these features are grouped into four groups. The first group has a feature, the second group adds a feature to the first, and the third group adds a feature to the second. The data set Sonar has a total of sixty features, which are divided into six groups. The first set of features is ten, and each subsequent set of features adds ten to the previous set of features.The order of addition is from large to small by $F_{i}$. The feature groups are formed in this way until the last feature group contains all the features. The classification results of some data sets are presented in Figure 1. Finally, the best feature group is determined according to the criteria of the highest classification accuracy and the least selected features. Table 3 shows the optimal number of features and the feature subsets selected for all data sets.

It can be seen from Figure 1 that the accuracy of all data sets fluctuates with the increase of the number of selected features. This means that many or few features do have a big impact on the accuracy of the data set. The few features make the model poorly learned. The excessive features will increase the training time and affect the efficiency. Moreover, the useless or redundant features will decrease the accuracy of the classifier. For the 14 selected data sets, the accuracy changes with the number of selected features, and the accuracy does not always increase with the number of features. Take the data set Sonar as an example: it has the highest accuracy at 40 features and decreases when the number of features continues to increase. As shown in Table 1 and Table 3, the number of features selected from some data sets (Iris, Vehicle, Diabetes, Australia) is close to that of the original data set when the accuracy reaches the highest. For example, the data set Diabetes has only one feature reduced compared to the original data set, while the data set Australia has only three features reduced compared to the original data set, although the number of features in these data sets is small. However, for some data sets (Farm Ads, Oclar, Arcene) with a large number of original features, the number of selected features at the time of reaching the highest accuracy is much lower than before after feature selection. For example, the data set Farm Ads decreases from 54,877 features to 1858 features, and the data set Oclar decreases from 3916 features to 435 features.

As the number of features decreases, the training time of the algorithm reduces significantly. As shown in Table 4, the training time of the data sets with a large number of features decreases significantly. For example, the training time of the Germen data set is reduced by nearly 15 min, and the training time of the CMC data set is reduced by nearly 7 min. Therefore, in the case of improved accuracy, the training time is greatly reduced, while the efficiency of the algorithm is greatly improved.

Accuracy is the most important evaluation index of the algorithm. It can be seen from Table 5 (optimal values are marked in bold) that the accuracy of most data sets has been greatly improved after feature selection. The accuracy is one to four percent higher. For example, the accuracy of data set Australia increases by 0.89%, and the accuracy of CMC increases by 4.26%. Some data sets have little or no improvement in accuracy as the data set itself has few features, and each of its features has a significant impact on the accuracy of classification. Therefore, the feature selection method reduces the number of features by only one or even no reduction, so its accuracy is improved relatively low.

### 4.4. Evaluation on DWPSO-SVM

In this part of the experiment, the DWPSO-SVM algorithm we proposed will be compared with the combination of the basic PSO algorithm and SVM, namely the PSO-SVM algorithm, and different evaluation indexes will be used for comparison. In addition to the PSO-SVM algorithm, the improved PSO-SVM algorithms and other types of algorithms related to SVM are also be compared. The UCI data sets listed in Table 1 are used for the experiment, and all features are selected by the FS-score method.

As shown in Table 6, experiments on 16 standard UCI data sets are conducted with feature selection in the improved PSO. The evaluation criteria we used are accuracy, TPR, and TNR. Ten experiments are performed for each data set to obtain the average value of all indicators. Among them, the values of TPR and TNR for data sets of three or more categories are expressed by N/A.

The results in Table 6 show that compared with the PSO algorithm, the DWPSO algorithm significantly improves the performance of the classifier on 16 data sets. Based on the accuracy, TPR, TNR, and other evaluation indexes, the proposed algorithm performs better than the basic PSO algorithm in most of the 16 data sets. For example, on the Vowel and Diabetes data sets, the accuracy of the indicators is 6.58% and 4.8% higher, respectively, than the algorithm before improved. For the Sonar data set, the TPR and TNR values for the DWPSO-SVM algorithm are 90% and 94.44%, respectively. Compared with the PSO-SVM algorithm, the results are respectively improved 5.48% and 1.11%. At the same time, its accuracy improves 4.77%. Although the accuracy is not high in some data sets, it also drops slightly for TPR and TNR. However, in general, compared with the PSO-SVM algorithm, the DWPSO-SVM algorithm has improved significantly in terms of classification accuracy. Meanwhile, the nonparametric statistical significance analysis is conducted to prove the strength of the proposed algorithm. As shown in Table 7, DWPSO-SVM is not significantly different from PSO-SVM at 0.05 level by the Kruskal–Wallis variance analysis.

In addition, other improved algorithms concerning the particle swarm inertia factor in recent years are also compared. It is combined with SVM in the same way as the DWPSO-SVM; other experimental conditions, parameters and configurations are the same. The DWPSO-SVM algorithm and other eight comparison algorithms are run on 12 data sets selected by the FS-score method, and its evaluation index is the accuracy of the classifier. The generation number and iteration number of all the compared algorithms have been listed in Table 8 (optimal values are marked in bold). As shown by the experimental results in Table 9 (optimal values are marked in bold), the accuracy of the proposed DWPSO-SVM algorithm is higher than that of other comparison algorithms in nine data sets. However, in the other three data sets, the classification accuracy is inferior to other comparison algorithms. For example, on the data sets Australia and Breast Cancer, the classification accuracy of the RAND-SVM method is the highest.

Table 10 (optimal values are marked in bold) shows the comparison between the DWPSO-SVM algorithm and other types of algorithms. Each algorithm adopts its default parameters and settings. The highest value of the classification accuracy on each data set has been shown in bold. It can be seen that in the nine data sets we adopted, the DWPSO-SVM algorithm has the highest accuracy.

Three data sets with different orders of magnitude have been selected for the memory experiment. For Iris, the data set with the least features, the memory occupied by the DWPSO-SVM method during training is 237 M due to the smallest number of samples. For Oclar, the data set with thousands of features, it takes up 2.32 GB of memory for training. For Farm Ads with about 50,000 features, it takes up 7.37 G of memory, which is almost the full memory. The time complexity of the algorithm is $O(n^{2})$, and the running time of the DWPSO-SVM algorithm on different data sets are is shown in Table 11. The spatial complexity of the algorithm is O(n).

**Figure 2:**
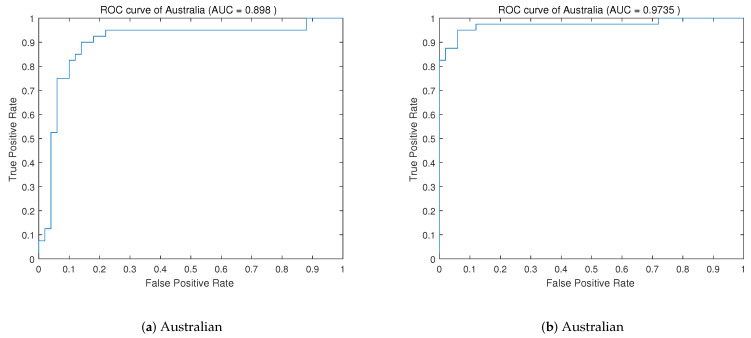
ROC curve of the Australia data set, (**a**) the ROC curve before improvement, (**b**) the ROC curve after improvement.

**Figure 3:**
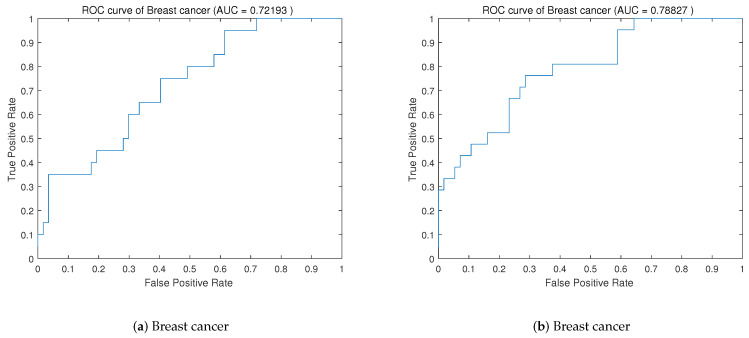
ROC curve of Breast cancer data set, (**a**) the ROC curve before improvement, (**b**) the ROC curve after improvement.

To evaluate the classifier’s ability to distinguish categories, the ROC curves of the data sets Australia and Breast Cancer are drawn. As shown in Figure 2 and Figure 3, the closer the curve is to the left and upper part of the coordinate axis, the better the classification ability of the classifier is. The larger the area of the curve is, the better the performance of the classifier. The area under the curve is the AUC value. We also calculate the AUC values of the Australian and Breast Cancer data sets under the DWPSO-SVM and PSO-SVM algorithms, which are illustrated in Figure 2 and Figure 3, respectively. From the perspective of the AUC value, the AUC value of the DWPSO-SVM algorithm is greater than that of the previous two data sets.

**Figure 4:**
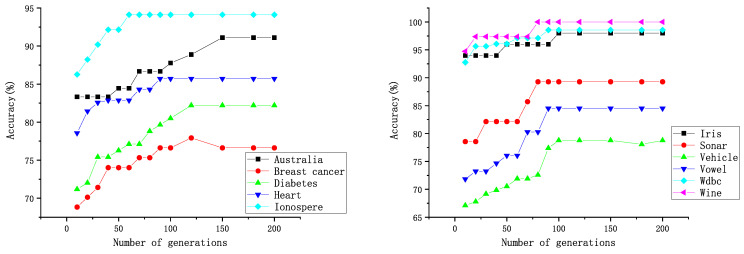
Running process of the DWPSO-SVM on 11 UCI data sets.

**Figure 5:**
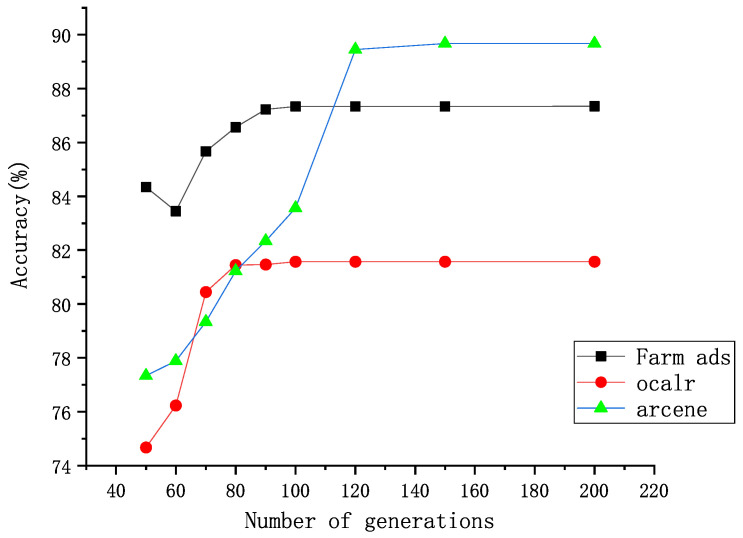
Running process of the DWPSO-SVM on 3 high-dimensional data sets.

The iterative process of the comparable algorithms is similar to that of DWPSO-SVM. Figure 4 and Figure 5 show the generation process of the DWPSO-SVM, in which the generation comes to stabilization when it reaches about 100 on almost all data sets. Table 8 shows the number of generations when other comparable algorithms reach convergence. As with the DWPSO-SVM, the comparable algorithms converge in about 100 generations. Table 11 shows the running time of DWPSO-SVM on different data sets. The DWPSO-SVM has some real-time performance on the data sets with a small number of features.

In general, the performance of our DWPSO-SVM classifier based on the PSO algorithm is better than that of the original elementary PSO algorithm in terms of the accuracy, TPR, TNR, and other evaluation criteria. Compared with other types of PSO and other swarm intelligence algorithms, the DWPSO-SVM has better classification accuracy on most data sets.

## 5. Conclusions

In this paper, a new feature selection method is proposed, which can be used for the feature selection of the multi-class data sets. The PSO-SVM algorithm is used to test the proposed feature selection method on some UCI data sets. The experimental results demonstrate that the proposed method is effective on the data sets with a large number of features. Compared with the MVO-SVM method [2], the proposed method performs better on some data sets, such as the Hear and Ionosphere data sets, with an accuracy rate 2.73% and 2.56% higher than MVO-SVM, respectively. Similarly, compared with the GA-SVM method [3], in the terms of the two indicators TNR and TPR, the proposed method also performs better on most data sets. For example, DWPSO is 17% higher on TPR and 1.33% higher on TNR than GA-SVM on the data sets Diabetes and Ionosphere. The classification accuracy is significantly improved, and the training time is significantly reduced.

Using the proposed feature selection method, an improved PSO algorithm is proposed to optimize the SVM parameters and RBF kernel parameters. By improving the variation of the inertia factor in the PSO algorithm, the population diversity in the later stage of the PSO algorithm can be increased to avoid premature convergence. In addition, the inertia factor can be adjusted and changed according to the actual situation. The experimental results show that compared with the hybrid algorithm of elementary particle swarm and SVM, the DWPSO-SVM is improved on all data sets in terms of accuracy, TPR, and TNR. Compared with other PSO algorithms and the meta-heuristic algorithms, our proposed algorithm is also optimal on most data sets, which proved that our improved algorithm has a significant effect on improving the accuracy of SVM.

The instability is the limitation of PSO, while the grid algorithm has good stability but poor efficiency. How to combine the advantages of these two algorithms needs to be explored in the next step. In fact, there are some redundant and invalid samples when applying the proposed feature selection algorithm. How to efficiently screen the redundant samples in the data set is also a problem that we want to focus on in the future. The training time can be decreased, since the accuracy of the DWPSO-SVM has satisfied almost all of the situations. For example, some scholars have proposed using the meta-learning method to reduce the training time. However, the meta-learning methods always make the accuracy of the classifier decrease. Therefore, we will attempt to reduce the training time of the classifier on the premise of maintaining accuracy and stability.

## Figures and Tables

**Table 1:** The data sets from the UCI repository.

Data Set	Features	Instances	Classes	Feature Characteristics	Data Set Characteristics
Australia	14	690	2	Categorical, Real	Multivariate, Time-Series
Breast cancer	9	277	2	Categorical	Multivariate
CMC	9	1473	3	Categorical, Integer	Multivariate
Diabetes	9	768	2	Categorical, Integer	Multivariate, Time-Series
Germen	24	1000	4	Integer, Real	Multivariate
Heart	14	270	2	Categorical, Integer, Real	Multivariate
Ionosphere	34	351	2	Integer, Real	Multivariate
Iris	4	150	2	Real	Multivariate
Dnatest	180	1186	3	Integer	Multivariate, Time-Series
Sonar	60	208	2	Integer	Multivariate, Text
Vehicle	18	846	4	Real	Multivariate, Integer
Vowel	3	871	6	N/A	Image
Wdbc	30	569	2	Real	Multivariate, Data-Generator
Wine	13	178	3	Integer, Real	Multivariate
Air	64	359	3	Real	Multivariate, Time-Series
Farm Ads	54,877	7143	2	N/A	Text
Oclar	3916	3916	5	Integer	Text
Arcene	10,000	900	2	Real	Multivariate

**Table 2:** The confusion matrix.

	Actual Class		
		1	−1
Predicted class	1	True Positive (TP)	False Negative (NP)
	−1	False Positive (FP)	True Negative (TN)

**Table 3:** The optimal feature subset.

Data Set	No. of Selected Features	Selected Features
Australia	11	2, 3, 4, 5, 6, 7, 8, 10, 12, 13, 14
CMC	5	1, 2, 3, 4, 8
Diabetes	7	1, 2, 3, 4, 5, 6, 8
Dnatest	28	5, 18, 44, 47, 50, 68, 72, 82, 83, 85, 90, 93, 97,
		99, 100, 105, 111, 120, 123, 126, 129, 150, 156, 162, 171,
		174, 180
Germen	15	1, 2, 3, 4, 5, 6, 7, 8, 9, 10, 11, 12, 13, 14, 17
Heart	12	1, 2, 3, 4, 5, 7, 8, 9, 10, 11, 12, 13
Iris	4	1, 2, 3, 4
Sonar	40	8, 9, 10, 11, 12, 13, 14, 15, 16, 17, 18, 19, 20,
		21, 22, 23, 24, 25, 26, 27, 28, 29, 30, 31, 32, 33, 34, 35, 36,
		37, 38, 39, 41, 42, 43, 44, 45, 46, 47, 48
Vehicle	10	1, 2, 3, 4, 7, 8, 9, 10, 11, 12, 13, 14, 16
Wdbc	25	1, 2, 3, 4, 5, 6, 7, 8, 9, 10, 11, 12, 13, 14, 19, 21,
		22, 23, 24, 25, 26, 27, 28, 29, 30
Wine	8	1, 3, 4, 5, 7, 10, 12, 13
Air	57	1, 2, 3, 4, 5, 6, 7, 15, 16, 17, 18, 19, 20, 21, 22,
		23, 24, 25, 26, 27, 28, 29, 30, 31, 32, 33, 34, 35, 36, 37,
		38, 39, 40, 41, 42, 43, 44, 45, 46, 47, 48, 49, 50, 51, 52,
		53, 54, 55, 56, 57, 58, 59, 60, 61, 62, 63, 64
Farm Ads	1858	N/A
Oclar	435	N/A
Arcene	175	N/A

**Table 4:** The comparison of the training time before and after feature selection.

Data Set	Training Time (s)		
	**Full Features**	**Selected Features**	**Percentage of Improvement (%)**
Australia	321	244	31.56
CMC	1382	987	40.02
Diabetes	305	295	3.38
Dnatest	4156	497	836.28
Germen	2245	1322	69.82
Heart	105	98	7.14
Iris	4	4	0.00
Sonar	62	44	40.91
Vehicle	782	610	28.20
Wdbc	478	430	11.16
Wine	54	46	17.39
Farm Ads	103,478	8758	1081.52
Oclar	8634	3287	162.67
Arcene	6438	1265	408.93

**Table 5:** The accuracy of PSO-SVM before and after feature selection.

Data Set		Accuracy (%)
	Full Features	Selected Features
Australia	83.56 ± 1.72	**84.45 ± 1.81**
CMC	49.59 ± 2.15	**53.85 ± 4.55**
Diabetes	75.08 ± 3.07	**77.54 ± 4.8**
Dnatest	54.92 ± 0.76	**90.91 ± 3.13**
Germen	49.50 ± 3.10	**53 ± 2.43**
Heart	80.57 ± 2.15	**83.57 ± 2.87**
Iris	95.4 ± 3.27	**97 ± 3.33**
Sonar	85.36 ± 4.28	**89.29 ± 4.25**
Vehicle	71.83 ± 2.94	**73.63 ± 4.13**
Wdbc	96.81 ± 2.44	**98.55 ± 2.83**
Wine	98.42 ± 2.22	**100 ± 0**
Farm Ads	47.48 ± 2.32	**77.78 ± 5.43**
Oclar	62.13 ± 7.18	**74.74 ± 4.23**
Arcene	67.37 ± 7.34	**81.54 ± 5.86**

**Table 6:** The comparison between the DWPSO-SVM (improved algorithm) and the PSO-SVM.

Data Set	DWPSO-SVM			PSO-SVM		
	TPR (%)	TNR (%)	Accuracy (%)	TPR (%)	TNR (%)	Accuracy (%)
Australia	88.89	91.11	87.41 ± 3.75	97.14	87.27	84.45 ± 1.81
Breast cancer	21.05	94.83	74.89 ± 4.49	25	92.98	71.43 ± 6.47
Diabetes	80	87.18	79.66 ± 4.56	56.76	87.65	74.86 ± 5.44
Germen	N/A	N/A	57.00 ± 5.29	N/A	N/A	57.00 ± 3.43
Heart	88.89	76.47	85.71 ± 2.86	83.78	75.76	82.86 ± 3.78
Ionosphere	90.48	100	93.46 ± 2.26	85	100	91.50 ± 2.26
Iris	N/A	N/A	98.67 ± 1.15	N/A	N/A	96.00 ± 2.00
Sonar	90	94.44	91.67 ± 2.06	84.62	93.33	86.90 ± 2.10
Vehicle	N/A	N/A	78.08 ± 4.31	N/A	N/A	75.11 ± 5.14
Vowel	N/A	N/A	84.51 ± 3.45	N/A	N/A	77.93 ± 5.33
Wdbc	N/A	N/A	99.03 ± 1.45	N/A	N/A	98.07 ± 0.84
Wine	N/A	N/A	100 ± 0	N/A	N/A	96.86 ± 0.12
CMC	N/A	N/A	54.38 ± 1.18	N/A	N/A	53.48 ± 1.94
Farm Ads	83.65	92.73	87.33 ± 3.42	77.89	84.46	79 ± 4.21
Oclar	N/A	N/A	81.46 ± 3.12	N/A	N/A	77.35 ± 2.45
Arcene	86.43	89.67	89.65 ± 4.65	83.83	85.94	83.75

**Table 7:** Kruskal–Wallis variance analysis.

Descriptive statistics						
	N	Min	Q1	Median	Q3	Max
DWPSO-SVM	13	54.38	76.485	85.71	96.065	100
PSO-SVM	13	53.48	73.145	82.86	93.75	98.07
**Rank**						
	N	Mean rank	Sum rank			
DWPSO-SVM	13	14.88462	193.5			
PSO-SVM	13	12.11538	157.5			
**Test statistics**						
	Chi-Square	DF	P > Chi-Square			
	0.85236	1	0.35588			

**Table 8:** Number of generations when the algorithm reaches convergence.

Data Set	DWPSO-SVM	RAND-SVM	SWISPSO-SVM	ALGPSO-SVM	AIWPSO-SVM	IIWPSO-SVM	IDWPSO-SVM	DSOPSO-SVM	PSO-A
Australia	93 ± 2.31	**90 ± 3.21**	103 ± 2.43	93 ± 1.32	92 ± 2.33	114 ± 3.42	115 ± 3.38	117 ± 3.12	110 ± 2.12
Breast cancer	77 ± 1.54	**67 ± 3.45**	86 ± 3.21	102 ± 4.33	130 ± 2.63	127 ± 2.39	132 ± 2.93	140 ± 2.45	128 ± 3.31
Heart	**87 ± 2.53**	97 ± 3.32	102 ± 3.73	80 ± 2.67	100 ± 3.56	94 ± 1.54	93 ± 1.56	88 ± 2.15	100 ± 3.21
Ionosphere	98 ± 2.12	**92 ± 2.13**	108 ± 3.11	100 ± 2.45	80 ± 2.15	94 ± 3.21	102 ± 3.18	94 ± 2.15	98 ± 2.16
Iris	**96 ± 2.41**	100 ± 1.12	118 ± 2.54	110 ± 1.15	103 ± 2.12	111 ± 2.15	115 ± 3.12	110 ± 2.15	102 ± 3.17
Sonar	**89 ± 3.12**	110 ± 1.27	95 ± 3.12	95 ± 2.17	114 ± 3.22	109 ± 1.24	**92 ± 2.12**	107 ± 2.87	100 ± 2.65
Vehicle	**79 ± 2.12**	90 ± 2.74	85 ± 3.52	90 ± 2.15	84 ± 1.28	82 ± 3.85	86 ± 2.16	85 ± 1.67	83 ± 1.42
Vowel	**84 ± 1.67**	97 ± 2.12	90 ± 1.43	100 ± 3.16	90 ± 3.12	95 ± 2.14	98 ± 1.43	104 ± 3.14	112 ± 3.16
Wdbc	**97 ± 2.15**	108 ± 4.12	100 ± 1.41	110 ± 2.15	113 ± 3.15	120 ± 1.34	118 ± 2.14	112 ± 1.45	117 ± 2.65
Wine	**100 ± 0.87**	120 ± 2.67	139 ± 3.56	108 ± 1.44	115 ± 1.78	112 ± 2.45	136 ± 3.12	114 ± 1.54	115 ± 1.34
Farm Ads	**87.33 ± 3.42**	77.43 ± 3.23	81.24 ± 2.34	63.34 ± 4.53	77.62 ± 4.29	63.45 ± 4.23	71.51 ± 3.62	83.56 ± 3.67	61.26 ± 4.18
Oclar	81.46 ± 3.12	**82.34 ± 4.34**	79.23 ± 3.24	53.34 ± 34	69.34 ± 3.61	52.45 ± 5.23	69.23 ± 6.16	75.57 ± 3.17	52.77 ± 3.67
Arcene	**89.65 ± 4.65**	81.34 ± 45	83.56 ± 5.23	66.56 ± 25	78.21 ± 1.66	71.23 ± 3.35	85. ± 4.32	87.23 ± 5.19	67.72 ± 3.82

**Table 9:** The accuracy comparison of the DWPSO-SVM algorithm and other types of the PSO-SVM.

Data Set	DWPSO-SVM	RAND-SVM	SWISPSO-SVM	ALGPSO-SVM	AIWPSO-SVM	IIWPSO-SVM	IDWPSO-SVM	DSOPSO-SVM	PSO-A
Australia	87.41 ± 3.57	**89.26 ± 2.23**	82.59 ± 6.3	85.56 ± 3.8	89.26 ± 3.01	83.33 ± 0.74	83.33 ± 2.54	82.59 ± 1.42	84.07 ± 3.06
Breast cancer	74.89 ± 4.49	**89.26 ± 2.81**	73.05 ± 2.08	70.56 ± 4.86	67.97 ± 1.82	67.97 ± 5.66	74.19 ± 3.53	73.76 ± 5.15	74.33 ± 4.51
Germen	**57.00 ± 5.29**	53.5 ± 4.35	47.00 ± 2.45	46.00 ± 5.08	49.50 ± 8.17	49.00 ± 4.72	50.00 ± 3.89	51.5 ± 4.35	57.00 ± 3.87
Heart	**85.71 ± 2.86**	85.67 ± 3.30	78.10 ± 3.60	77.14 ± 5.15	82.38 ± 4.59	84.29 ± 5.15	83.33 ± 3.60	84.29 ± 1.43	82.86 ± 1.43
Ionosphere	93.46 ± 2.26	95.42 ± 1.13	90.20 ± 1.96	96.07 ± 3.40	**96.73 ± 1.13**	95.42 ± 3.00	92.16 ± 5.19	95.42 ± 3.00	94.12 ± 1.96
Iris	**98.67 ± 1.15**	96.67 ± 1.15	93.33 ± 4.62	94.67 ± 1.15	96.67 ± 1.15	95.33 ± 0.97	96.00 ± 2.31	95.33 ± 1.00	96.00 ± 1.33
Sonar	**91.67 ± 2.06**	85.71 ± 7.14	88.10 ± 5.45	88.10 ± 7.43	85.71 ± 3.56	85.71 ± 7.14	89.28 ± 3.57	84.52 ± 5.46	86.90 ± 14.4
Vehicle	**78.08 ± 4.31**	71.49 ± 3.77	74.32 ± 0.68	71.69 ± 3.38	74.66 ± 4.17	71.58 ± 7.88	72.60 ± 4.17	71.46 ± 1.43	76.25 ± 3.09
Vowel	**84.51 ± 3.45**	76.06 ± 5.63	78.87 ± 6.14	76.06 ± 2.44	82.16 ± 2.93	80.28 ± 2.82	76.69 ± 4.30	77.46 ± 6.14	75.12 ± 1.63
Wdbc	**99.03 ± 1.45**	97.10 ± 2.51	98.06 ± 1.67	97.59 ± 0.84	97.10 ± 1.45	96.14 ± 2.21	96.62 ± 3.02	97.10 ± 1.45	96.45 ± 0.84
Wine	**100 ± 0**	98.25 ± 1.52	97.37 ± 2.63	99.12 ± 1.52	99.12 ± 1.52	99.12 ± 1.36	97.37 ± 2.51	99.12 ± 1.52	99.11 ± 1.52
CMC	**54.38 ± 1.18**	53.15 ± 1.29	52.81 ± 4.17	52.38 ± 1.12	50.92 ± 2.60	51.10 ± 0.68	49.74 ± 1.27	49.45 ± 1.73	50.74 ± 4.94

**Table 10:** The accuracy comparison between the DWPSO-SVM algorithm and other types of algorithms.

Data Set	DWPSO-SVM	GA-SVM	DA	GOA	SCA	SSA	WOA	BAT
Australia	**87.74**	68.6	68.6	66.67	71.02	71.01	67.45	67.45
Diabetes	**79.66**	71.49	64.47	71.93	78.95	60.09	79.82	70.18
Germen	57	**80**	78.67	72	79.67	73.67	79	78.33
Heart	**85.71**	74.21	66.67	56.79	78.31	75.31	67.8	75.31
Iris	**98.67**	95.55	95.55	97.78	95.56	93.33	95.56	100
Sonar	**86.9**	80	83.33	86.33	83.33	78.33	86.33	78.33
Vehicle	78.08	80.95	79.76	43.25	79.76	82.54	79.37	**84.13**
Vowel	84.51	98.72	**100**	98.72	99.36	100	99.36	98.72
Wine	**100**	62.74	72.55	76.47	84.31	86.27	62.75	94.12

**Table 11:** The running time of DWPSO-SVM on different data sets.

Data Set	DWPSO-SVM Running Time (s)
Austtalia	266 ± 12
CMC	1122 ± 33
Diabetes	324 ± 23
Dnatest	505 ± 45
Germen	1445 ± 126
Heart	100 ± 5
Iris	4 ± 1
Sonar	36 ± 3
Vehicle	593 ± 44
Wdbc	465 ± 14
Wine	56 ± 8
Farm Ads	8426 ± 445
Oclar	3354 ± 321
Arcene	2478 ± 435

## Data Availability

https://archive.ics.uci.edu.
